# Retracted: Management Practice, and Adherence and Its Contributing Factors among Patients with Chronic Kidney Disease at Tikur Anbessa Specialized Hospital: A Hospital Based Cross-Sectional Study

**DOI:** 10.1155/2019/9043263

**Published:** 2019-02-05

**Authors:** International Journal of Nephrology

*International Journal of Nephrology* has retracted the article titled “Management Practice, and Adherence and Its Contributing Factors among Patients with Chronic Kidney Disease at Tikur Anbessa Specialized Hospital: A Hospital Based Cross-Sectional Study” [1]. The article was previously published as Kefale B, Tadesse Y, Alebachew M, Engidawork E (2018) Management practice, and adherence and its contributing factors among patients with chronic kidney disease at Tikur Anbessa Specialized Hospital: A hospital-based cross-sectional study. PLoS ONE 13(7): e0200415. https://doi.org/10.1371/journal.pone.0200415 [2].
